# Leveraging research in early cognitive development for application and impact

**DOI:** 10.3389/fdpys.2026.1745860

**Published:** 2026-03-08

**Authors:** John Colombo, Kimberly Cuevas, D. Jill Shaddy

**Affiliations:** 1 Schiefelbusch Institute for Life Span Studies and Department of Psychology, University of Kansas, Lawrence, KS, United States; 2 Department of Psychological Sciences, University of Connecticut, Storrs, CT, United States; 3 Department of Dietetics and Nutrition, University of Kansas Medical Center, Kansas City, KS, United States

**Keywords:** biobehavioral markers, clinical trials, cognitive assessment, cognitive development, individual differences, infancy, longitudinal research, translational research

## Abstract

Research in cognitive development has generally been aligned with the explication and understanding of basic mechanisms underlying change in mental development over time. We suggest here that work done in the measurement and assessment of granular cognitive functions be leveraged toward translational or applied work in order to realize enhanced impact and influence. Specifically, we propose the use of cognitive measures or performance on laboratory-based cognitive tasks as outcomes in clinical trials, as biobehavioral markers for developmental risk or disorders, and perhaps even as intervention methods *per se*. We argue that this strategy can address changing pressures in both public and private sponsorship as well as contribute to building stronger public support for research endeavors in the field.

## Introduction

Broadly speaking, we conduct research for two reasons. First, we design and implement scientific inquiry and analysis to try and understand the phenomena and processes that bear on ourselves and on the world around us. Within the realm of cognitive development, for example, we seek to understand the nature of our cognitive abilities, when they emerge, how they emerge, and the various forces that might enhance or diminish them. Indeed, much of the work done in the area of cognitive development over the past century has been conducted with these aims in mind; prominent examples include the longstanding debate over nature and nurture as determinants of neurodevelopment (Gottlieb, 2014), the dynamic interplay between the emergence of various skills over the course of development (e.g., Masten and Cicchetti, 2010), and the emergence of sophisticated and complex cognition (i.e., executive function) from the integration of lower-order cognitive components (Banich, 2009; Colombo and Cheatham, 2006; Cuevas et al., 2017; Garon et al., 2008).

We also do research, however, to seek scientifically-based solutions to more immediate or proximal problems in various domains. Using research in this way has long been a point of discussion in the field of psychology (Miller, 1969; Sanford, 1965), although at least for some, the degree to which behavioral or psychological research has had tenable impacts on larger societal problems is a matter of debate (Halverson et al., 2021). For the most part, while the field of cognitive development has often paid lip service to such goals (we have all read articles or reviewed grant proposals that place the justification for basic research as contributing to the identification of developmental risk or to the development of interventions for clinical populations), these intentions have been only sparsely realized. This is especially true with respect to cognitive development in infancy and early childhood, where promise for such progress would seem to have the greatest potential. As such, we would argue that the application of cognitive development to solving societal problems represents a relatively new direction for this field and represents the focus of this paper.

It is our position that the field of cognitive development has certainly matured to the point that innovative use in applied contexts would appear to be more feasible, impactful, and timely. To be sure, there have been efforts to bring state-of-the-art ideas about cognitive development to fruition in applied contexts (e.g., Carlson, 2023), we still too often find public considerations of applications of cognitive development to be mired reanalyses of Piagetian theory and in the debate over innate vs. acquired skills (Rochat, 2023), or in recitations of simple developmental milestones and common-sense child-rearing practices (Crotty et al., 2023).

In this brief perspective, we seek to leverage our experience in using measures of cognitive development in applied contexts to elaborate on this as a new direction for emphasis within the discipline. In those experiences, the application of cognitive development research in impactful contexts comes with some nuanced and unforeseen issues that we hope to explicate here. We will end with a discussion of some of the implications of this emphasis for training in the field as well as for the mindset of scientists working in the field.

## Examples of leveraging cognitive development in applied designs

### Using cognitive measures as clinical trial outcomes

For over a decade now, federal sponsors such as the National Institutes of Health (NIH) have promoted a research agenda emphasizing more proximal impact (Collins, 2010; Sampat, 2012; Zerhouni, 2006) and the promulgation of clinical trials (Agarwal and Tu, 2022).

Indeed, the development of developmental “toolboxes” initiated by the NIH (e.g., Han et al., 2025; Shields et al., 2020) represents a response to the desire to move granular, laboratory-based tasks of neurodevelopment and neurocognition into the realm of standardized measures for use in more practical applications, including clinical trials to assess the feasibility, safety, and efficacy of interventions designed to improve developmental outcomes.

Given the repeatedly-demonstrated disproportionate influence of early life on the trajectory of development over the lifespan (Bornstein, 1989; Colombo, 1982), the theoretical case for such intervention to either enhance typical development (Ramey and Ramey, 1998) or alter the course of development in children at risk for delay or disability (Guralnick, 2011) is compelling. In the operationalization of evaluating the efficacy of such interventions through a randomized clinical trial, one must choose how and when to assess their effects. In deference to an audience that is unfamiliar with the principles of development or developmental assessment, there is often pressure to choose some standardized outcome that is widely known or accepted outside of the behavioral sciences (for example, pediatric or educational fields).

However, such standardized outcomes are typically, by definition, measures of global status, and such measures carry limitations. First, the measures themselves may assess constructs that are, in fact, resistant to change; for example, some of the initial criticisms of Early Head Start programs were rooted in the fact that the program was unable to produce permanent increases in children’s performance on intelligence (i.e., IQ) tests (Zigler and Styfco, 1994) when multiple studies have shown that altering IQ scores is enormously difficult (see Howe, 1998). Second, global developmental measures may tap individual differences that are irrelevant to ultimate developmental outcomes; for example, performance on the Bayley Scales (Bayley, 2006) in early development relies heavily on sensorimotor function, which has been repeatedly shown to be uncorrelated with later cognition, at least within the range of typically-developing infants and children (Colombo, 1993; McCall, 1981). Finally, since performance on such measures is usually expressed in terms of a score derived from performance across several domains or a subscore within a domain across different component tasks (e.g., a cognitive score calculated across sensory, attention, memory, language, and executive skills) they may be insensitive to the effects of an intervention if the intervention has effects specific to a particular cognitive subsystem or brain area (Colombo, 2001; Colombo and Carlson, 2012; Wainwright and Colombo, 2006).

As an example of the adaptation of lab-based cognitive measures in clinical trials, we offer a history of over a dozen randomized clinical trials since the 1990s from our University of Kansas laboratory investigating the effects of prenatal and postnatal nutritional supplementation on cognitive development. Most of this work has examined the effects of omega-3 and/or omega-6 long-chain polyunsaturated fatty acids (Colombo et al., 2004, 2011, 2013, 2016) but we have also used this strategy to examine the effects of prebiotics (Colombo et al., 2021), trace elements like zinc and copper (Colombo et al., 2014), and most recently, the combination of nutritive elements present in the membrane that surrounds the large fat globules in mammalian milk (Colombo et al., 2023; Li et al., 2019). Following the implementation of these nutritional interventions either provided to mothers during pregnancy or fed to infants during the first year, children were enrolled in longitudinal follow-ups in which they were periodically assessed on various measures of cognition across developmentally sensitive periods for each, including visual habituation during the first year, measures of attention and memory during the second year, and then various measures of emergent executive function afterwards. In many cases, the efficacy of these interventions was successfully documented, and in some cases, we actually observed long-term changes in language and IQ. The value of using early cognitive assessments in these trials cannot be overstated; they afforded sponsors a compelling basis for continuing the longitudinal schedule to document long-term effects.

This work did not come without difficulties in collaborating across disciplines; the principles of cognitive development and assessment that we routinely take for granted in developmental science are not necessarily intuitive to others who work outside that sphere. Indeed, the scientists and health care professionals with whom we collaborated initially came to these trials with several assumptions.

The first assumption was that the effect of an early intervention (in this specific case, any nutrient or supplement) should be evident with any cognitive assay; a corollary of this assumption was the expectation that any effects on a measure would persist over the long term and also be evident in other assays. We often attributed this assumption to an intuitive hypothesis that cognitive development is driven by some underlying factor that perfectly maps to developmental outcomes into childhood (and beyond). Of course, cognitive development is far more complex than that, as it is comprised of numerous dissociable components (Cuevas and Sheya, 2019; Kail, 2004; Michel et al., 2015) that likely influence each other through developmental cascades (Masten and Cicchetti, 2010), and early exposures to different environmental conditions may differentially affect these components (Boucher et al., 2014).

Second, given our collaborators’ familiarity and experience with global developmental tests like the Bayley Scales, Mullen Scales (Mullen, 1995), or other standardized assessments of early performance, questions constantly arose as to why a single measure could not be used at all ages of the longitudinal schedule. Again, the complications of developmental assessment is not always intuitive for non-developmentalists; the notions that (a) different manifestations of neurocognitive development will not be appropriate at all ages, or (b) that the effect of an intervention might be evident on a particular measure at one age, but not on that measure later on, or (c) that effect may then be seen on more sophisticated measures at later ages were neither familiar nor comfortable. Indeed, we often saw these patterns of outcomes in our research on omega-3 fatty acids; for example, the positive effects of supplementation with docosahexaenoic acid were initially seen in terms of acceleration in the development of visual acuity during the first year; however, that advantage typically “washed out” later in infancy (see Cheatham et al., 2006), only to have the positive effects of supplementation re-emerge on measures of visual attention (Colombo et al., 2011) or executive function later on and through the preschool years (Colombo et al., 2013). While such “washout” (or “fadeout,” see Bailey et al., 2020) can be attributable to many reasons, our data show this particular phenomenon to be driven by the sensitivity of chosen outcome measures.

Finally, our collaborators and sponsors often presumed that if the intervention were effective, its results would be seen across all ages; that is, only a single outcome point of assessment would be necessary (this also aligned with the strategy of reducing the cost of the trial). We often referred to examples of rapid development during early life to convince our colleagues of the value of a strategy in which different cognitive outcomes were assessed across different ages at which those outcome measures were appropriate (e.g., measures of visual attention in the first year, followed by assessments of memory in the second year, and then executive function in the third). This was particularly critical, because in many cases, we were only able to demonstrate the efficacy of an early intervention in terms of a changed trajectory in a cognitive measure (Colombo et al., 2004, 2013, 2016). Such a finding would, of course, not be possible if the outcome in a clinical trial were limited to a single “snapshot” assessment at a primary endpoint (see, for example, Colombo et al., 2016).

The point most relevant for this paper from this section is that our understanding of early cognition allowed us to leverage laboratory assessments of early cognition that had been used previously in basic-science studies of attention, perception, and executive function with infants, toddlers, and preschoolers to prompt changes in practice and industry that would not have occurred if the research had relied solely on the more broadly recognized global measures. We were able to validate proper dosage, document effects of nutrition as clinically proven, and contribute to changes in the formulation of several products, affecting (literally) millions of infants on a global scale.

### Using cognitive development as biobehavioral markers

Since the inception of the field of developmental science, measures of cognitive development have been used as markers in the early detection of intellectual or developmental delay or disability; this was in fact the fundamental premise of the design and implementation of assays such as the Bayley and Gesell scales in the first half of the 20th century (Colombo, 1993). During the 1970s and 1980s, the reported success of the Fagan Test of Infant Intelligence (see Benasich and Bejar, 1992) spurred interest in the predictive validity of various indices of infant cognition. Although the promise of the Fagan Test was never fulfilled (Tasbihsazan et al., 2003), other more specific indices measured in infancy were shown to be contributors to later cognitive outcomes (Bornstein and Colombo, 2012; McCall and Carriger, 1993), if in indirect ways (Bornstein et al., 2006, 2013).

However, more recently, reports on the use of more granular laboratory-based measures of early cognition (e.g., measures of visual habituation, free-play attention, inhibition, discrimination learning) have been used in concert with either later prediction of neurodevelopmental status or as biobehavioral markers of developmental delay or atypical development. This approach has been used successfully for some time in the realm of cognitive aging (e.g., Albert, 1996; Unsworth et al., 2015; Whelan et al., 2022), but has not necessarily been a focal point with respect to the use of cognitive measures.

There are countless examples of investigations of the use of cognitive development measures in examining the correlates of intellectual/developmental disability conditions such as autism spectrum disorders (Johnson et al., 2021), attention deficit/hyperactivity disorder (ADHD; Castellanos et al., 2006; Cheung et al., 2016), or more readily identifiable conditions such as Down Syndrome (e.g., Edgin, 2013) or Fragile X Syndrome (Huddleston et al., 2014), but there are relatively few examples of the use of early cognitive or neurocognitive measures to prospectively identify later developmental differences or neurodevelopmental conditions.

The use of cognitive development measures here would prove useful for more nuanced developmental profiles, such as learning disabilities (Barnes et al., 2020; Johnson et al., 2010) or ADHD (Nichols and Waschbusch, 2004). While individual differences in the measures themselves might predict delay or disability, it is also the case that profiles of interrelationships between tasks (Houwen et al., 2016) or developmental trajectories (Tullo et al., 2023) could be predictive as well.

### Cognitive development measures as interventions

Early cognition emerges through continuous coaction among neural, behavioral, and environmental systems and in the context of intricately linked and mutually influential changes across physical, biological, social, and other domains (Gottlieb, 2007; Michel et al., 2015). From this perspective, repeated exposure to measures designed to assess cognitive function may, under some circumstances, also serve to promote development, thereby blurring the traditional line between assessment and intervention.

Examples of this potential come from research on the training of infant attention, where shifting the spotlight of information processing may serve as a causal mechanism for broader cognitive development. Classic work by Rose and colleagues (Jankowski et al., 2001) established that, at least in the short-term, salient cues could alter infants’ characteristic looking patterns, shifting long-lookers toward more efficient sampling behaviors associated with enhanced learning. More recent studies using gaze-contingent attention training with eye tracking have extended these findings, showing short-term gains in attentional control and regulation among typically developing infants (Forssman and Wass, 2018; Wass et al., 2011). This approach has also been adapted in small-scale studies with infants at risk (e.g., ADHD, prematurity, autism, socioeconomic adversity), providing proof of concept, though with mixed evidence for effectiveness (Goodwin et al., 2021; Perra et al., 2021). Although evidence of durable or generalized effects remains limited, these studies underscore the potential of targeted cognitive measures to induce meaningful short-term change in early regulatory systems.

A broader set of contingency-based interventions further illustrates the active role that sensorimotor learning experiences can play in shaping development (e.g., Sullivan and Lewis, 1990). As summarized in a recent systematic review of 17 studies (Inamdar et al., 2022), paradigms such as operant mobile tasks, high-amplitude sucking, and the “sticky mittens” intervention allow infants to experience direct relations between their actions and environmental outcomes. Engagement in these experiences has been associated with gains in motor control, exploratory behavior, language, and cognition among typically developing infants, with emerging evidence for benefits in those with neurodevelopmental conditions or environmental risks. Although findings are mixed and many studies are small in scale and short in duration, there is clear need for larger, high-quality randomized clinical trials. At the same time, the success of these approaches in naturalistic or home-based contexts—often with parent participation—highlights their translational promise.

Taken together, this work suggests that cognitive assessments can serve dual purposes, functioning both as analytic tools and as structured learning experiences that promote adaptive change. Through mechanisms such as attention training and contingency learning, these approaches may strengthen foundational “learning to learn” capacities, influencing multiple domains of development through cascading effects. At the same time, evidence for durable far transfer—particularly for interventions targeting higher-order cognitive functions, such as executive control, at later developmental periods—remains limited (e.g., Doebel, 2020; Kassai et al., 2019). Early modulation of basic cognitive processes may therefore offer greater leverage for downstream development. Although still an emerging area of inquiry, identifying the ecological and developmental bounds under which cognitive measures function most effectively as interventions represents a critical direction for future research and a natural extension of basic research into applied practice, offering a promising avenue for advancing both mechanistic understanding and societal impact.

## Requisite conditions for application

We are advocating for the field to move more strongly toward the use of the products of the field of cognitive development in applied settings. The potential for enhancing the impact of the field may be greatest in the expanded utilization of measures developed for academic/basic research questions in more applied settings. The adoption of the use of cognitive development measures for applied purposes is different from the basic-science mindset that guides most cognitive development research; it requires some conceptual adjustments in the conduct of the work, and additional perspectives in training if this is to be promulgated for graduate students in the field. Most of these changes involve a shift in the emphasis on cognitive-development phenomena per se to a fundamental consideration of individual differences.

### Commitment to measuring meaningful individual differences

#### A focus on longitudinal design

In adopting cognitive measures for clinical trials or biobehavioral markers, a fundamental assumption is that the measures we develop and implement must reflect indices that are meaningful reflections of the individual infant or child. This requires a direct focus on establishing the psychometric properties of the measures we use. This includes documentation of test-retest reliability across both brief and long intervals (in developmental contexts, reliability across different ages has been previously referred to as stability; see Colombo and Fagen, 2014) as well as the evaluation of the normative developmental course. All of this would necessarily be based on some form of longitudinal work; while such work is more common now than it has been in the past (Grammer et al., 2013), it seems safe to say that it has not necessarily been a point of emphasis within cognitive developmental studies. For training students, this likely would require the addition of measurement theory in graduate curricula as well as an emphasis on the logistics of planning longitudinal work and analysis of longitudinal data (and its related problems; see Menard, 2007).

#### Establishing process-product associations

A corollary to the emphasis on psychometric properties is the need to validate that what one is measuring (i.e., the product) actually represents the cognitive mechanism or skill that one presumes to underlie the measure itself (i.e., the cognitive process). This notion was best expressed over a half century ago in a classic paper by Underwood (1975). The argument put forth is that theories based on group (i.e., nomothetic) data should be subject to tests based on individual (i.e., idiographic) data. The fundamental concept is that if a theory about group-based phenomena was correct, then it should be confirmable (or refutable) by looking at correlations within individuals between the proposed cognitive process and the measured product of that process. While this concept is seemingly obvious, it has routinely been neglected within the field of cognitive development (and, to be fair, in cognitive psychology as well).

### Interdisciplinary collaboration

As a final point, in all of our endeavors in applying cognitive developmental principles to translational research, we have worked with individuals outside of the realm of developmental science. Developing and maintaining these collaborative working relationships has not been a turnkey enterprise, as each discipline has its distinct culture and knowledge base. Those collaborations work best when both parties are willing to step out of their comfort zones and learn about each other’s science in more than a superficial manner. We have had to devote time to learning about early intervention strategies, the metabolic pathways for nutrients, the rules for conduct of clinical trials, and other aspects of implementation science. Through all of this, a sense of humility, mutual respect, and patience in the process of mutual education has served us well and contributed directly to the success and productivity of the science.

## Summary and conclusions

We have argued that there have long been two traditions in developmental science, one that seeks to explicate and understand the basic mechanisms underlying change in human behavior over time, and another that has been more closely associated with addressing practical and applied problems surrounding the identification and amelioration of risk and atypical conditions affecting developmental outcome. While research in cognitive development (and especially early cognitive development) has generally been more aligned with the former, we have suggested that work in cognitive development might look to shift its focus or direction toward a more translational approach.

We have identified three areas in which the field might leverage expertise in the assessment of cognitive functions (again, especially in early cognition) to enhance impact and influence: the use of cognitive measures or performance on laboratory-based cognitive tasks as (a) outcomes in clinical trials, (b) biobehavioral markers for developmental risk or disorders, and (c) as intervention methods in and of themselves, based on the notion that repeated exposure to the tasks or measures might result in improvements that either persist across age, or filter out to affect other cognitive abilities or skills.

It is our position that pursuit of this type of strategy in the field of cognitive development would work to address changing pressures from public and private sponsors seeking greater practical or translational impacts in the behavioral sciences, and the shift toward these types of work might also contribute to building stronger public support for the research effort in this area nationally and internationally.

## Data Availability

The original contributions presented in this perspective are included in the article, further inquiries can be directed to the corresponding author/s.
